# Utilizing deep learning models in an intelligent spiral drawing classification system for Parkinson’s disease classification

**DOI:** 10.3389/fmed.2024.1453743

**Published:** 2024-09-04

**Authors:** Nesren Farhah

**Affiliations:** Department of Health Informatics, College of Health Sciences, Saudi Electronic University, Riyadh, Saudi Arabia

**Keywords:** Parkinson’s disease, transfer learning models, deep learning, hand drawing, E-health

## Abstract

**Introduction:**

Parkinson’s disease (PD) is a neurodegenerative illness that impairs normal human movement. The primary cause of PD is the deficiency of dopamine in the human brain. PD also leads to several other challenges, including insomnia, eating disturbances, excessive sleepiness, fluctuations in blood pressure, sexual dysfunction, and other issues.

**Methods:**

The suggested system is an extremely promising technological strategy that may help medical professionals provide accurate and unbiased disease diagnoses. This is accomplished by utilizing significant and unique traits taken from spiral drawings connected to Parkinson’s disease. While PD cannot be cured, early administration of drugs may significantly improve the condition of a patient with PD. An expeditious and accurate clinical classification of PD ensures that efficacious therapeutic interventions can commence promptly, potentially impeding the advancement of the disease and enhancing the quality of life for both patients and their caregivers. Transfer learning models have been applied to diagnose PD by analyzing important and distinctive characteristics extracted from hand-drawn spirals. The studies were carried out in conjunction with a comparison analysis employing 102 spiral drawings. This work enhances current research by analyzing the effectiveness of transfer learning models, including VGG19, InceptionV3, ResNet50v2, and DenseNet169, for identifying PD using hand-drawn spirals.

**Results:**

Transfer machine learning models demonstrate highly encouraging outcomes in providing a precise and reliable classification of PD. Actual results demonstrate that the InceptionV3 model achieved a high accuracy of 89% when learning from spiral drawing images and had a superior receiver operating characteristic (ROC) curve value of 95%.

**Discussion:**

The comparison results suggest that PD identification using these models is currently at the forefront of PD research. The dataset will be enlarged, transfer learning strategies will be investigated, and the system’s integration into a comprehensive Parkinson’s monitoring and evaluation platform will be looked into as future research areas. The results of this study could lead to a better quality of life for Parkinson’s sufferers, individualized treatment, and an early classification.

## 1 Introduction

Parkinson’s disease (PD) is a chronic deteriorating illness that primarily affects the motor system of the central nervous system. Its indications often manifest gradually, and as the disease progresses, non-motor indications become more prevalent. The primary indications are tremors, stiffness, bradykinesia, and gait disturbances. PD may also result in dysphoria, apprehension, sleep disturbances, sensory impairments, and alterations in behavior. Environmental factors and genetic inheritance are significant contributors to the development of PD (1, 2).

In 2019, a World Health Organization research reported that approximately 8.5 million individuals are diagnosed with PD (3). The prevalence of this condition increases with age, with only 4% of afflicted persons younger than 50 years old. PD is a highly prevalent neurological disorder worldwide, ranking as the second most common condition after Alzheimer’s disease. It affects a significant number of people, as evidenced by the data from sources (4, 5). Currently, therapists have limitations in effectively treating the symptoms of this condition as interventions are still in their early stages (6). The main tool used to determine a PD classification (PDD) is the patient’s medicinal past; however, such classification remains uncertain (3). Thus, it is critical to offer a simple and reliable method for detecting this disease in order to save time and money on invasive classification and treatment (7, 8).

Patients with PD may exhibit a broad variety of non-motor symptoms, including mood disorders and depression, among others. These symptoms, including language and other relevant aspects, may manifest in the patient’s facial expressions (9). The present study aims to analyze the effect of PD on both motor and non-motor abilities by applying handwriting modeling methodologies, with a special focus on spirals. This study seeks to fill a current knowledge gap by exploring the potential of spiral drawing as a tool for PD assessment.

Spiral drawing is a sophisticated and intricate motor skill that requires coordination. Consequently, it is regarded as an accurate evaluation of motor function. The Motion Rating Scale and its subcategory, The Unified PD Rating Scale (UPDRS-III), are the predominant and universally acknowledged rating scales for assessing PD. PD impacts a range of bodily processes, including speaking, handwriting, walking, and coordination, all of which are classified as motor functions. Various methods for measuring motor decline and non-motor biomarkers have been proposed to assess the severity of PD, which is considered a motor condition resulting from neurodegeneration. Both the classification and intensive care of PD are expensive and challenging because of two primary factors: (1) the inconvenience faced by caregivers in transporting the patient to the clinic and (2) the need for skilled medical professionals to conduct physical examinations and make diagnoses based on their observations. Clinical invasive techniques are only accessible at the early stage of the disease, and they carry risks and require considerable resources, especially in underdeveloped regions of the world. These techniques are only beneficial if early classification is achieved (10, 11).

At present, there is no accurate standard for making an objective finding of PD. When a non-specialist makes the classification, the likelihood of a mistake increases dramatically. There is a 20% chance of making a wrong classification in such instances (12). The accuracy of the classification is improved by carefully analyzing the main indications, which include tremors, bradykinesia, and stiffness. Having said that, physician bias may creep into clinical assessments. Medical choice support systems are attracting interest for their capability to enhance objectivity and facilitate early classification. An early identification of PD will enable the development of tailored interventions for people with PD (13, 14). A crucial objective in the study of neurodegenerative illnesses is to discover precise biomarkers (15). Within the literature, several research have been conducted to diagnose PD by analyzing speech. These studies (16–18) mostly use sustained vowels and natural speech for diagnostic purposes. Motor symptoms may also be identified and monitored by analyzing patients’ motions and gait (19, 20).

Several techniques have been created to examine the handwriting of patients with PD (21). Both static and dynamic characteristics are intriguing, including factors such as speed and the lowering of pen pressure throughout the handwriting (22). Numerous recent review studies have been published (23, 24). The legibility of an individual’s handwriting is influenced by their visual acuity, writing technique, and linguistic proficiency, resulting in significant differences across individuals. A viable substitute for handwriting is the use of illustrations. Deep learning (DL) models have greatly revolutionized biomedical and medical image analysis (25). DL approaches have been applied in different domains, including segmentation, detection, classification, and classification (11), owing to their exceptional capability to extract sophisticated features, leading to enhanced accuracy in illness categorization. This may mostly be ascribed to their remarkable ability to generalize. Convolutional neural networks (CNNs) have been crucial in promoting the progress of the medical imaging field, achieving notable success in several medical image classification tasks (19, 20).

### 1.1 Main contribution

Spiral drawing is a sophisticated and intricate motor skill that requires coordination. Accordingly, it is regarded as an accurate evaluation of motor function and an initial examination for early indications of PD. This article proposes a method for PDD by analyzing spiral drawings and employing transfer learning models. The method categorizes an individual as either healthy or diagnoses them with PD based on their spiral drawing. A spiral drawing produced by a healthy individual will closely resemble a typical spiral form. By contrast, a spiral created by an individual with PD will exhibit significant deviation from a flawless spiral form and appear twisted because of the individual’s sluggish motor movements and diminished synchronization between the hand and the brain.

## 2 Related works

Drotar and colleagues planned the utilization of a feature selection algorithm and support vector machine (SVM) approach to analyze the handwriting of patients with PD (26, 27). Their study is one of the first efforts to analyze the results of hand motions in the air or on a surface for diagnosing motor disorders associated with neurodegenerative illnesses. The findings revealed that these motions have a significant influence on the evaluation of handwriting and achieve a prediction accuracy of 85.61% (26). The work featured the PaHaW handwriting database, which was created by having individuals with PD complete eight distinct handwriting challenges, one being the Archimedean spiral. Basnin et al. (27) demonstrated their approach by using deep transfer learning, achieving a testing accuracy of 91.36%. The research only used a dataset consisting of 800 hand-drawn spiral pictures. Das et al. (28) investigated a sophisticated technique for identifying PD using pictures that were hand-drawn by the patients. The authors combined discrete wavelet transform coefficients with histograms of oriented gradient data to enhance the accuracy of detection rate. They revealed the effectiveness of integrating these methods to extract pertinent information and identify vital coefficients, resulting in improved accuracy in disease detection using machine learning techniques. They specifically highlighted the efficacy of random forest (RF) and SVM approaches when applied to spiral pattern features of images.

Researchers have discovered that studying handwriting or hand drawings is a more efficient method for identifying PD (29). Shaban (30) advocated for the use of a meticulously adjusted VGG19 model that applies spiral and wave handwriting patterns to diagnose conditions. The dataset used was of limited size and comprised 102 wave photos and 102 spiral images. Data augmentation, such as applying picture rotation, was used to alleviate the problem of model overfitting. After implementing 10-fold cross-validation, the CNN model demonstrated impressive accuracies of 88 and 89% for the wave and spiral pictures, respectively. Megha Kamble et al. (31) proposed a comprehensive examination of the static and dynamic spirals created by people with Parkinson’s disease. To do this, we extracted kinematic characteristics related to movement in the air and on the surface from data files created for 25 patients and 15 healthy controls. We utilized mathematical models for this purpose. Gil-Martín (32) this study contributes to the ongoing endeavor by examining a convolutional neural network (CNN) for the purpose of detecting PD based on drawing gestures. The analysis was conducted with a publicly available dataset: Digitized graphics are utilized to create spiral drawings for Parkinson’s disease. Donalto Impedovo et al. (33) have proposed handwriting as a robust indicator for the development of a diagnostic tool for Parkinson’s disease. The authors have applied a machine learning classification framework to the PaHaW dataset and achieved high specificity performance scores. Marta San Lucianol et al. (34) proposed the utilization of spiral drawing for computerized analysis of PD, as digitized spirals demonstrate a correlation with motor scores. The indices that are generated or calculated that have a correlation with the overall execution of a spiral include severity, shape, and kinematic irregularity. Kinematic irregularity includes second order smoothness and first order zero crossing. Other indices include tightness, mean speed, and variability of spiral width. Theyazn H. H. Aldhyani et al. (10) study makes a contribution by utilizing deep learning models to diagnose PD using photos of spiral and wave drawings. Manju Singh et al. (35) aims to provide a method for detecting PD utilizing spiral sketching and convolutional neural networks (CNN). The core concept is to examine an individual’s spiral drawings and categorize them as either indicative of good health or indicative of Parkinson’s disease. The spiral doodles produced by individuals in good health bear a striking resemblance to conventional helical forms. Table 1 presents a concise summary of the key attributes of prior studies on PD identification using drawings and other datasets.

**Table 1:** Overview of the current state of the art in employing various types of publicly available datasets based on artificial intelligence techniques.

Authors	Datasets	Approaches	Object of study
Vanegas et al. (42)	Parkinson’s dataset in EGG	Decision tree approaches	For this study, authors employed machine learning techniques to create a model that can accurately detect the most significant indicators from the EEG spectra during visual stimulation. The purpose of this model is to aid in the classification of PD.
Oh et al. (43)	Parkinson’s dataset in EGG	CNN model	This study utilized the electroencephalogram (EEG) data of twenty individuals with PD and twenty individuals without PD. An established CNN architecture consisting of thirteen layers effectively eliminates the requirement for traditional feature representation stages.
Prasuhn et al. (44)	Parkinson’s dataset in using MRI images	SVM approach	The proposed work suggests utilizing computer-aided methods and a highly reproducible method, as opposed to manually segmenting Substantia nigra (SN) to enhance the dependability and precision of Diffusion Tensor Imaging (DTI) of the measurements employed for categorisation.
Rasheed et al. (45)	Parkinson’s dataset in using voice	BPVAM	This study presents two classification algorithms aimed at enhancing the accuracy of identifying PD cases based on voice measures. Initially, implemented the BPVAM algorithm, which is a variable adaptive moment-based backpropagation algorithm of artificial neural networks (ANN).
Gunduz et al. (46)	Parkinson’s dataset in using voice	GB model	This study presents two frameworks utilizing CNNs method to accurately classify PD by analyzing sets of vocal (voice) data. Both frameworks are used to combine different feature sets, but they differ in how they combine these sets.
Pdisher et al. (47)	Parkinson’s dataset collected using sensor device	CNN model	Employed DL techniques to categorize motion data obtained from a solitary IMU sensor worn on the wrist, which was recorded in unstructured settings. In order to validate the results, patients were followed by a specialist in movement disorders, and their motor condition was assessed regularly and without active participation every minute.
Taliki et al. (48)	Parkinson’s dataset in using sensory	Random forest	This article explores instances of misclassification and presents a proposed system for obtaining a second opinion. The system relies on wearable sensors and artificial intelligence. To address this issue, authors developed several standardized tasks and collected movement data using wearable sensors worn by persons diagnosed with PD other extrapyramidal illnesses.
Shaban et al. (30)	Parkinson’s dataset using hand drawing	DL-based VGG16	This work explores the application of a fine-tuned VGG-19 model to screen for PD using a Kaggle handwriting dataset. The study involves conducting experiments to test the effectiveness of this approach. The dataset consisted of 102 wave and 102 spiral handwriting patterns.
Robin (49)	Parkinson’s dataset using hand drawing	RestNet50	Developing RestNet50 to detect PD using of 102 of 102 wave and 102 spiral
Stpete_ishii (50)	Parkinson’s dataset using hand drawing	CNN model	Developing online for classification of PD by using spiral images
Shaban et al. (30)	Handwriting dataset (same dataset)	CNN model	Developing online for classification of PD by using spiral images
Adrian (36)	Parkinson’s dataset using hand drawing	CNN model	Developing online for classification of PD by using spiral images

## 3 Materials and methods

**Figure 1:**
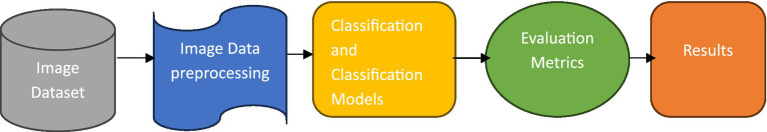
Framework of the proposed methodology.

This section details the planned methodology applied to develop a PDD system based on DL techniques, specifically designed to detect PD from features extracted from spiral drawing images. This methodology includes dataset collection, data preprocessing, DL classification models, evaluation metrics, and results analysis. The framework of this methodology is shown in Figure 1.

### 3.1 Dataset collection

**Figure 2:**
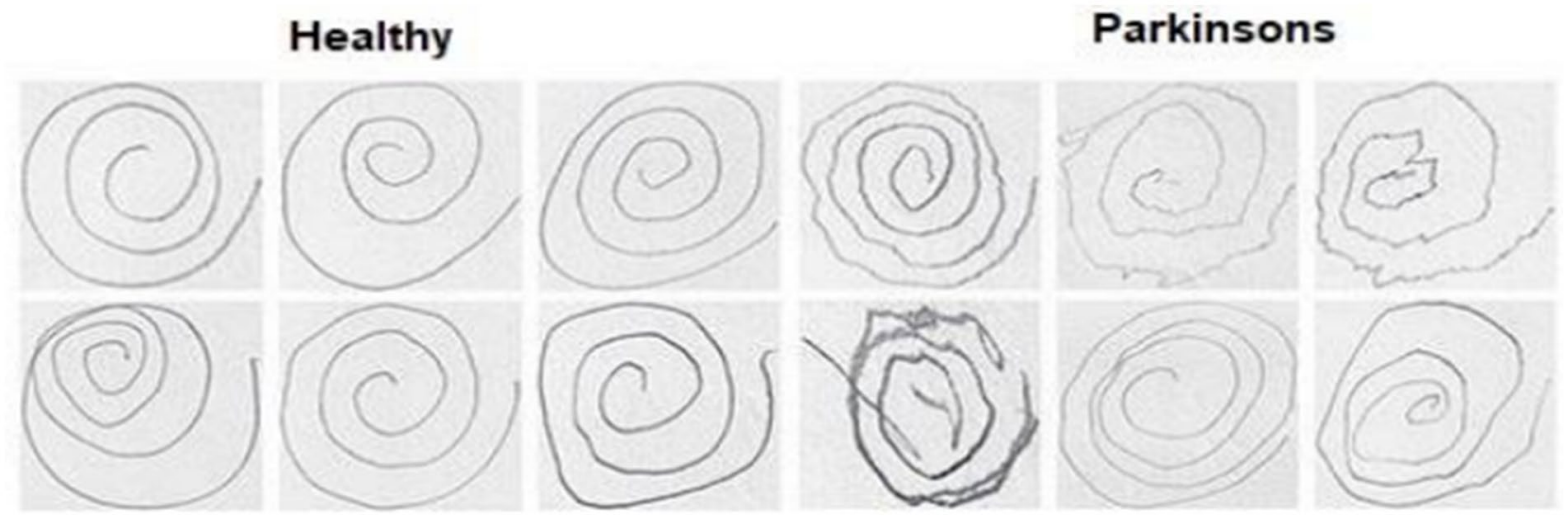
Samples of spiral drawing images dataset.

For our experimental study, we employed a dataset of spiral drawing images obtained from the Kaggle platform. This dataset, which was created by Adriano et al. (36) based on the NIATS of the Federal University, includes digital records of 102 spiral image samples, with 51 from Parkinson’s disease patients (PDP) and 51 from healthy persons. The images have been pre-split into a training set and a testing set (Figure 2).

### 3.2 Data preprocessing

**Figure 3:**
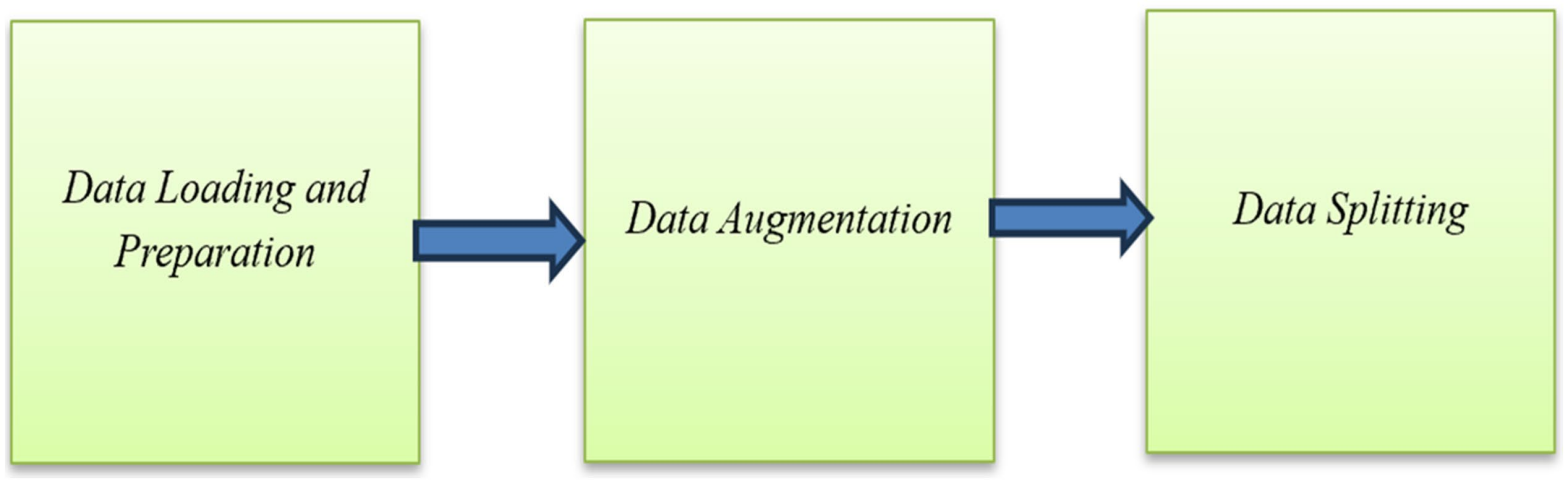
Preprocessing steps.

For our experimental work on PDD using drawn spiral images, we utilized a comprehensive dataset from the Kaggle platform. This dataset includes digital drawings from 51 PDPs and 51 healthy individuals. The processing steps are presented in Figure 3.

#### 3.2.1 Data loading and preparation

The dataset was divided into two classes: “healthy” and “parkinson.” Each image was resized to 100 × 100 pixels and converted to array format for consistency. Labels were encoded into binary format, where “healthy” was labeled as 0 and “parkinson” as 1. This preparation step ensured uniform input data for the model.

#### 3.2.2 Data augmentation

To increase the diversity and robustness of the training dataset, we applied data augmentation techniques using the Image Data Generator module, including rotation, shifting, and flipping of images. We likewise introduced variations that prevent overfitting and enhance the model’s capability to generalize to novel, unnoticed image data.

#### 3.2.3 Data splitting

In this step, we split the dataset into a training set and a testing set using an 80-20 split ratio. This stratification ensures a balanced representation of both classes in the training and testing phases.

#### 3.2.4 Normalization and label encoding

The pixel values of the images were standardized to the range [0, 1] to expedite the training process and improve model performance. Additionally, the labels were one-hot encoded to facilitate categorical classification.

### 3.3 Diagnoses and classification models

For the classification and classification of drawn spiral images into the “Parkinson” and “healthy” classes, we applied several advanced CNN architectures, including VGG19, InceptionV3, ResNet50v2, and DenseNet169. These models were pre-trained on the ImageNet dataset, which comprises over 14 million images across 1,000 categories. ImageNet provides a robust foundation for transfer learning due to its diverse range of visual concepts, although it does not inherently include clinical images.

#### 3.3.1 VGG19 model

**Figure 4:**
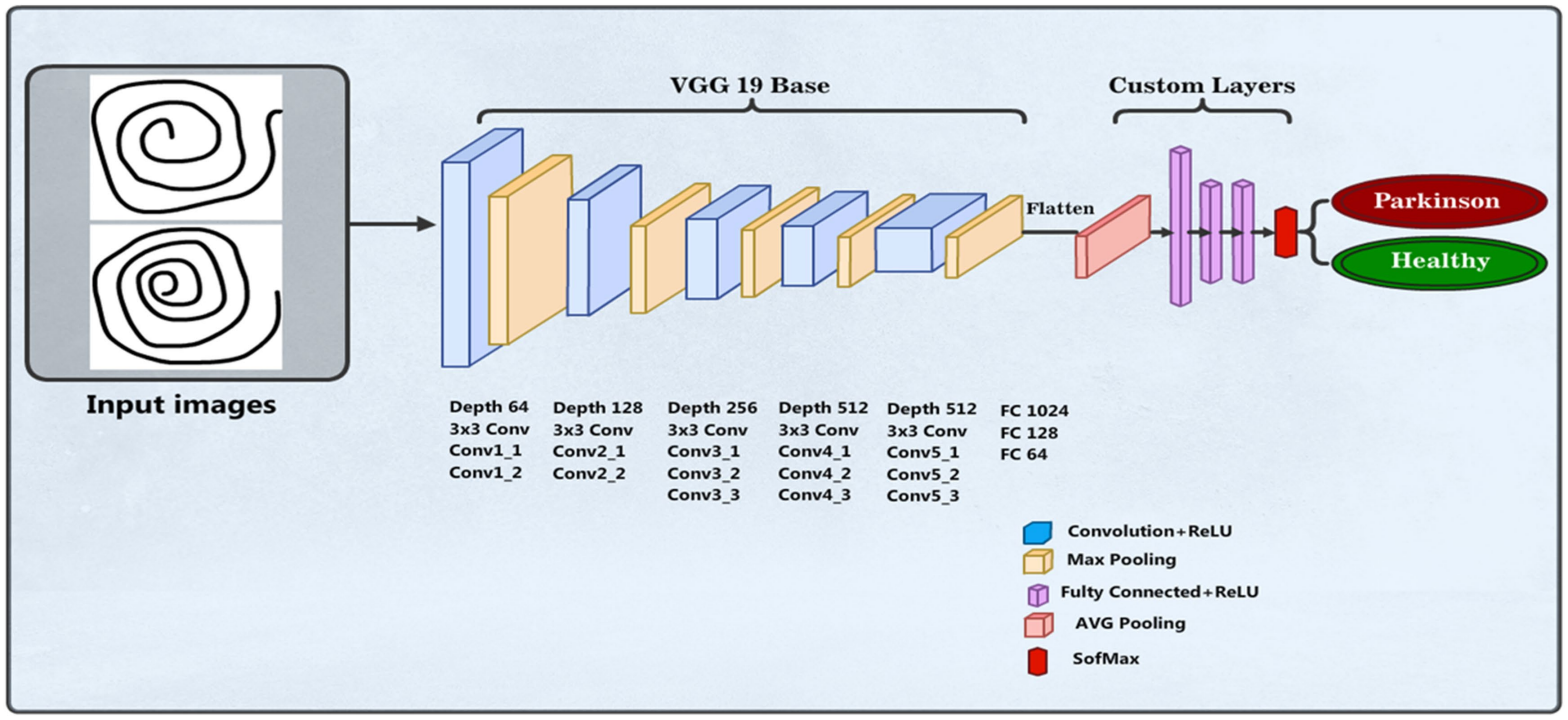
Structure of the VGG19 model.

We employed a CNN using the pre-trained VGG19 model to identify PD (37). The input layer accepts images resized to 100 × 100 pixels with three color channels. The model, pre-trained on the ImageNet dataset and excluding its top categorization layer, assists as a feature mining with average pooling. This is followed by a custom dense layer with 64 units and ReLU activation to introduce nonlinearity. The final layer is a dense output layer with 2 units and softmax activation, designed for binary classification between healthy individuals and PDPs. The model is compiled with the Adam optimizer, where categorical cross-entropy is the loss function and accuracy is the assessment metric. The data training process was conducted over 50 epochs with a batch size of 16 samples in each iteration, utilizing augmented training data. Figure 4 shows the VGG19 model architecture. The parameters of the VGG19 model are presented in Table 2.

**Table 2:** Summary of the VGG19 model parameters.

Layer	Parameters
Input Layer	(100, 100, 3)
VGG19 Base Model	Pre-trained on ImageNet, include_top = False, average pooling
Dense Layer	64 units, ReLU activation
Output Layer	2 units, Softmax activation
Optimizer	Adam
Loss Function	Categorical Cross-entropy
Metrics	Accuracy
No. of Epochs	50
Batch Size Used	16

#### 3.3.2 InceptionV3 model

**Figure 5:**
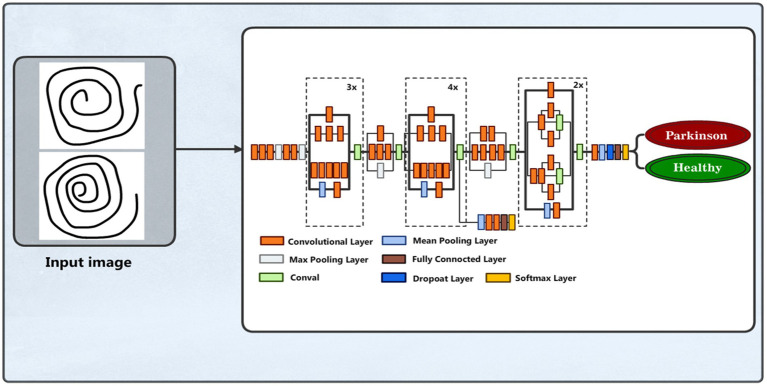
Inception model structure.

We also employed the pre-trained InceptionV3 model (38), whose inception modules are well known for their effective multi-scale feature extraction capabilities for PD detection by analyzing spiral drawing image features. Images with three color channels and a resizing of 100 × 100 pixels are accepted by the input layer. With average pooling, the InceptionV3 model functions as the feature extractor, omitting its top classification layer. To add nonlinearity, a bespoke dense layer with 128 units and a ReLU activation function is applied. The last layer is a dense output layer for binary classification among individuals without PD and those with the condition. Figure 5 depicts the Inception model structure.

InceptionV3 has two units in the output layer to represent the dataset classes, namely, Parkinson and Healthy, as well as softmax activation applied for the classification task. The Adam optimizer is used to create the model. Model training is carried out using a batch size of 32 utilizing augmented training data across 50 epochs. Table 3 summarizes the inception model parameters and their values used to develop and implement the model.

**Table 3:** Summary of the Inception model parameters.

Layer	Parameters
Input layer	(100, 100, 3)
Dense layer	128 units, ReLU activation
Output layer	2 units, Softmax activation
Optimizer	Adam
Loss function	Categorical Cross-entropy
Metrics	Accuracy
No. of epochs	50
Batch size used	32

#### 3.3.3 DenseNet169 model

**Figure 6:**
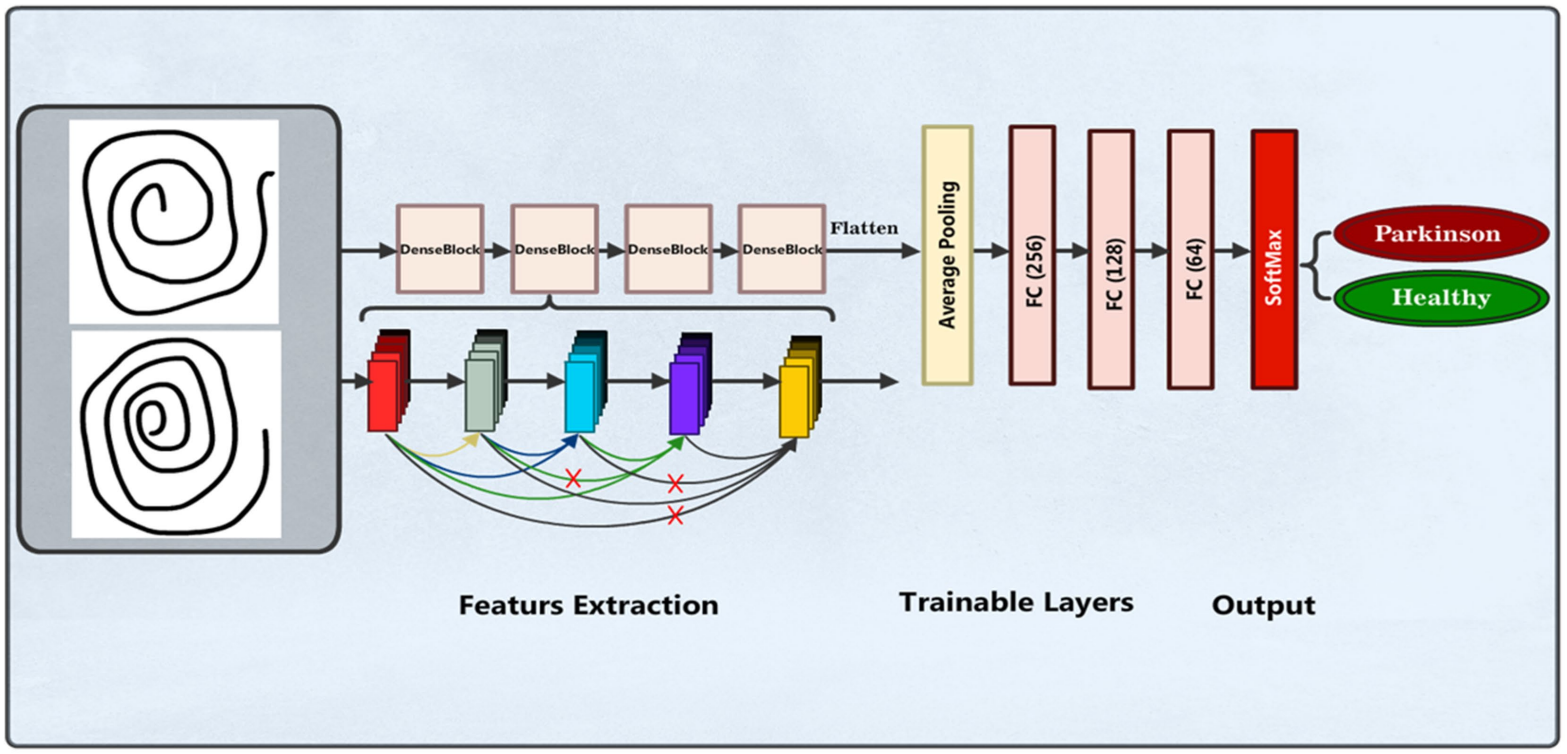
DenseNet169 model structure.

We also applied the pre-trained DenseNet169 (39, 40) model for PD detection and classification based on spiral drawing image features. This model is known for having a dense pattern of connectivity that promotes improved feature reuse and maximum information flow across layers. Images with three color channels and a resizing of 224 × 224 pixels are accepted by the input layer. With average pooling, the pre-trained DenseNet169 model functions as the feature extractor, omitting its top classification layer. A bespoke dense layer with 128 units and a ReLU activation function is applied to add nonlinearity. Figure 6 illustrates the DenseNet169 model structure.

The final layer is a dense output layer used for binary classification between individuals without PD and those with the condition. Also known as the output or last layer, this layer has two units to represent the dataset classes and uses a Softmax activation function to calculate the probability of each sample being either PPD or Healthy. The model utilizes accuracy as the evaluation measure, categorical cross-entropy as the loss function, and the Adam optimizer for training. Table 4 presents the summary of the model parameters used.

**Table 4:** Summary of the DenseNet169 model parameters.

Layer	Parameters
Input layer	(224, 224, 3)
DenseNet169 base model	Pre-trained on ImageNet, include_top = False, average pooling
Dense layer	128 units, ReLU activation
Output layer	2 units, Softmax activation
Optimizer	Adam
Loss function	Categorical cross-entropy
Metrics	Accuracy
No. of epochs	50
Batch size used	16

#### 3.3.4 ResNet50v2 model

**Figure 7:**
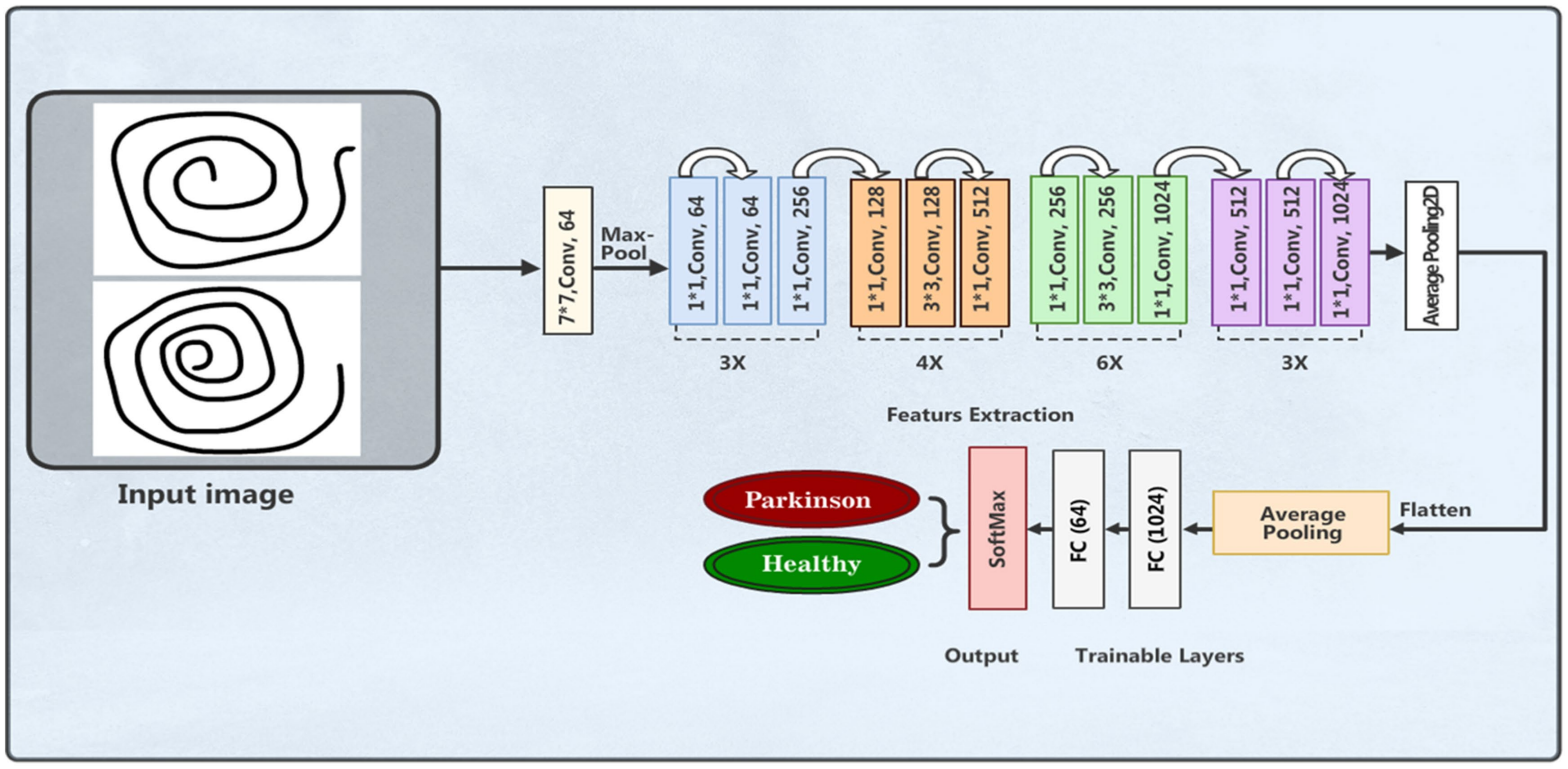
ResNet50 model architecture.

A DL framework called residual network (ResNet) was presented by Kaiming He et al. (41). The capability of this architecture to effectively train deep neural networks has attracted huge interest. The main breakthrough in ResNet is the use of residual connections, or skip connections, which improve gradient flow and lessen the problem of vanishing gradients. The residual blocks make up the bulk of the ResNet architecture. These blocks are made up of multiple convolutional layers, an activation function (usually ReLU), and batch normalization. The skip link, which enables the direct addition of the block’s input to its output, is what distinguishes a residual block. This method enhances gradient flow during backpropagation and helps the network learn residual functions. We applied the ResNet50v2 model structure in our experimental work for PD detection and classification based the features of spiral drawing images. The images were scaled to 224 × 224 pixels with three color channels an can be loaded into the input layer. The feature extractor with average pooling is the pre-trained ResNet50v2 model without its top classification layer. Nonlinearity is added by adding a customized dense layer with 128 neurons and a ReLU activation function. Figure 7 depicts the model architecture.

The final layer is an output layer with two neurons and softmax activation function for binary classification of patients with PD and healthy people. Categorical cross-entropy is used as the loss function, accuracy is the assessment measure, and the model is assembled based on the Adam optimizer. Using supplemented training data, the training process was run across 50 epochs with a batch size of 32. Table 5 outlines the model parameters used.

**Table 5:** Summary of the ResNet50 model parameters.

Layer	Parameters
Input layer	(224, 224, 3)
ResNet50v2 base model	Pre-trained on ImageNet, include_top = False, average pooling
Dense layer	128 units, ReLU activation
Output layer	2 units, Softmax activation
Optimizer	Adam
Loss function	Categorical Cross-entropy
Metrics	Accuracy
No. of epochs	50
Batch size used	32

To evaluate the models’ performance on our current dataset, we first trained these pre-trained models on the spiral image dataset before testing them. We recorded performance metrics such as accuracy, precision, recall, and F1-score.

### 3.4 Evaluation metrics

Assessing the performance and testing results obtained by the proposed DL models, namely, VGG19, DenseNet169, Inception, and ResNet50v2, are crucial for gauging the effectiveness of the models. Several metrics are used to quantify performance, including precision, recall, accuracy, F1-score, and ROC curve, which are calculated from the confusion matrix. The evaluation measures provide an alternative perspective on the advantages and disadvantages of the model.


(1) $$\mathrm{Accuracy}=\frac{TP+TN}{FP+FN+TP+TN}\times 100$$

(2) $$F1-\mathrm{score}=2*\frac{\mathrm{precision}\times \mathrm{Recall}\;}{\mathrm{precision}+\mathrm{Recall}\;}\;x100\%$$

(3) $$\mathrm{Precision}=\frac{\mathrm{True}\;\mathrm{Positives}\;}{\mathrm{True}\;\mathrm{Positives}+\mathrm{False}\;\mathrm{Positives}\;}x100\%$$

(4) $$\mathrm{Recall}=\mathrm{Sensitivity}=\frac{\mathrm{True}\;\mathrm{Positive}\;}{\mathrm{True}\;\mathrm{Positive}+\mathrm{False}\;\mathrm{Negatives}}x100\%$$

## 4 Experimental results

This section reports the findings obtained from various experiments carried out for PD recognition and classification using various DL models, namely, VGG19, ResNet50, InceptionV3, and DenseNet169. Each model was assessed based on its ability to accurately categorize spiral drawn images from patients with PD and healthy individuals.

### 4.1 Testing results of the VGG19 model

As revealed in Table 6 below, an overall accuracy of 72% is shown in the testing classification results for PD recognition utilizing the VGG19 model. With a recall of 86% and precision of 60% for Parkinson’s cases, the model successfully recognized the majority of Parkinson’s cases with a small number of false positives.

**Table 6:** Testing classification results of the VGG19 model.

	Precision %	Recall %	F1-score%	Support	Accuracy%
Parkinson	60	86	71	7	72
Healthy	88	64	74	11	
Macro average	74	75	72	18	

**Figure 8:**
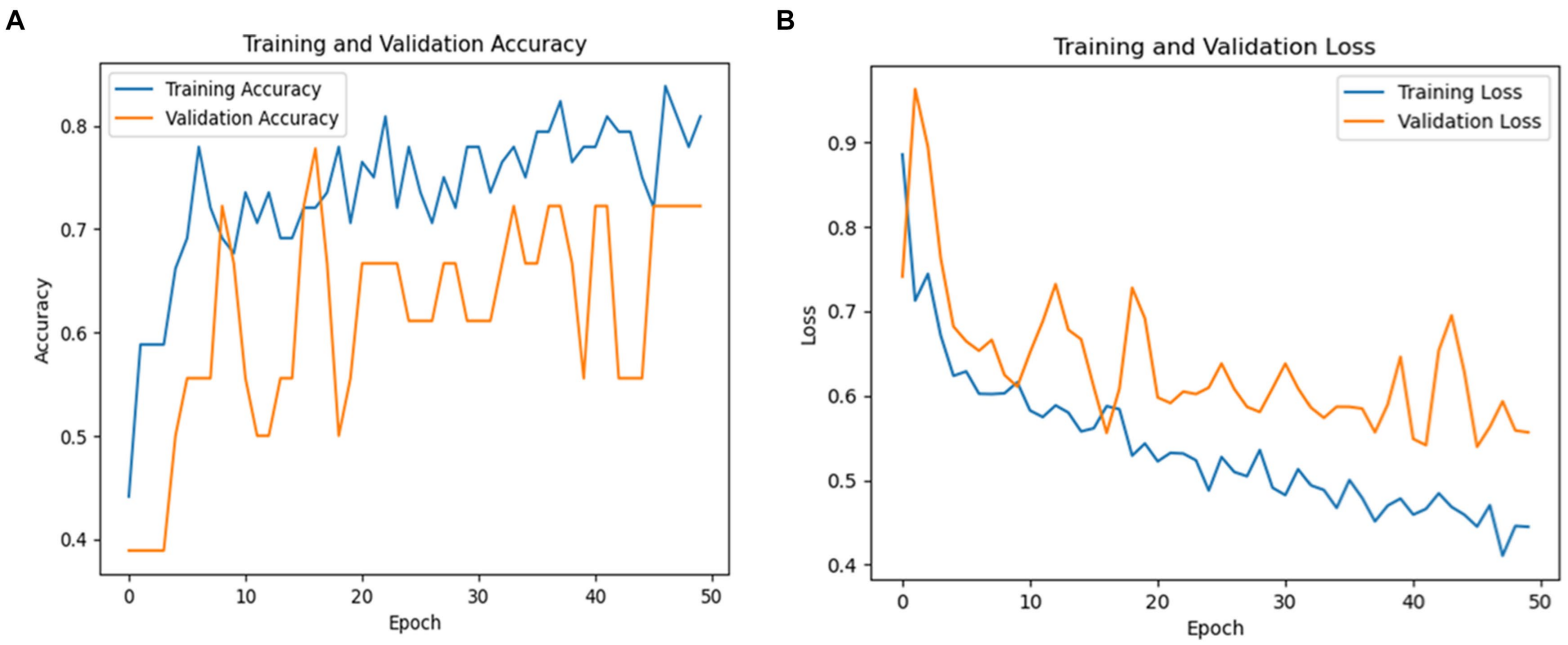
**(A)** Validation and training accuracies of the model, **(B)** model loss, and **(C)** AUC of the VGG19 model.

Recall was 64% and precision was 88% for healthy persons, indicating a higher classification accuracy for healthy cases but with some false negatives. For Parkinson’s patients, the F1-score was 71, while for healthy cases it was 74. The macro averages for precision, recall, and F1-score were 74, 75, and 72%, respectively. These findings point to areas where the model might be improved to lower classification mistakes while also demonstrating how well it detects PD. Figure 8 shows a graphical representation of the performance results for the VGG19 model.

Figure 8A illustrates the validation and training accuracies of the model over 50 epochs, presenting how well it learned to distinguish between Parkinson’s and healthy cases. Figure 8B presents the model’s loss over the training period, indicating the reduction in prediction error as training progressed. Figure 8C depicts the area under the curve (AUC) of the VGG19 model, providing a quantity of the model’s capacity to distinguish between the two classes with an AUC value of 81% The AUC is a valuable metric for evaluating the overall results of the classification model.

### 4.2 Testing results of the inception v3 model

The testing classification findings utilizing the InceptionV3 model for PD identification are given in Table 7. The InceptionV3 model attained an overall accuracy of 89%. For Parkinson’s cases, the model achieved a precision of 78% and a recall of 100%, indicating it accurately recognized all true Parkinson’s occurrences but included some false positives. For healthy individuals, the precision was 100% and the recall was 82%, showing exceptional precision but missing some real healthy examples. The F1-score for PD was 88%, and for healthy persons, it was 90%.

**Table 7:** Testing classification results of the InceptionV3 model.

	Precision %	Recall %	F1-score%	Support	Accuracy%
Parkinson	78	100	88	7	**89**
Healthy	100	82	90	11	
Macro average	91	89	89	18	

**Figure 9:**
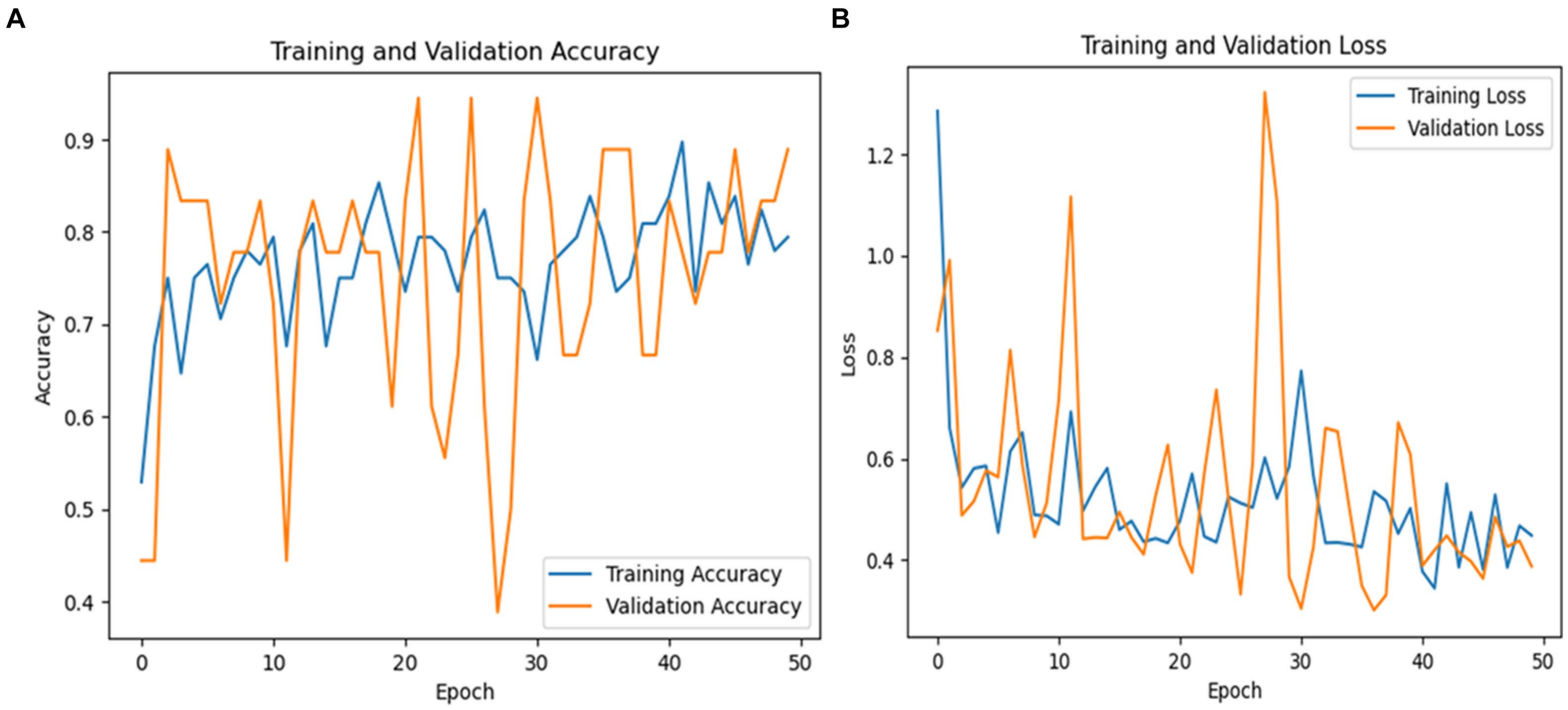
**(A)** Validation and training accuracies of the model, **(B)** model loss of the InceptionV3 model.

The overall averages of the metrics are 91% for precision, 89% for recall, and 89% for F1-score, demonstrating the balanced performance of the model across both classes. These results suggest that InceptionV3 is highly effective for PD detection, particularly excelling in correctly identifying true cases of the disease. Figure 9 shows a graphical representation of the performance results for the Inceptionv3 model.

Figure 9A shows the validation and training accuracies, which started at 55% and ended at 79% for training and the validation started at 45% and ended at 89%. The significant improvement from the initial to the final epoch indicates effective learning. Figure 9B illustrates the model’s loss over the training period, with a notable reduction from an initial loss of 1.2840 to a final loss of 0.4486 for training and 0.3879 for validation, indicating the increased ability of the model to make accurate predictions. Figure 9C depicts the AUC of the InceptionV3 model, which reached an impressive value of 95, demonstrating the robust discriminative ability of the model between Parkinson’s and healthy cases.

### 4.3 Testing results of the ResNet50v2 model

This subsection presents the outcomes of our experiments utilizing the ResNet50v2 model for the detection and classification of Parkinson’s Disease (PD). The model achieved an overall accuracy of 80%. For instances of Parkinson’s, the ResNet50v2 model exhibited a precision of 79% and a recall of 92%. This indicates that the model correctly identified 92% of Parkinson’s cases within the testing set, though it produced some false positives. In contrast, for healthy individuals, the model attained a precision of 83% and a recall of 62%, signifying a reasonable accuracy in classifying healthy cases but missing some true healthy instances. The F1-scores were 85% for Parkinson’s cases and 71% for healthy cases. The testing classification performance of the ResNet50V2 model is summarized in Table 8.

**Table 8:** Testing classification results of the ResNet50v2 model.

	Precision %	Recall %	F1-score %	Support	Accuracy %
Parkinson	79	92	85	12	**80**
Healthy	83	62	71	8	
Macro average	81	77	78	20	

**Figure 10:**
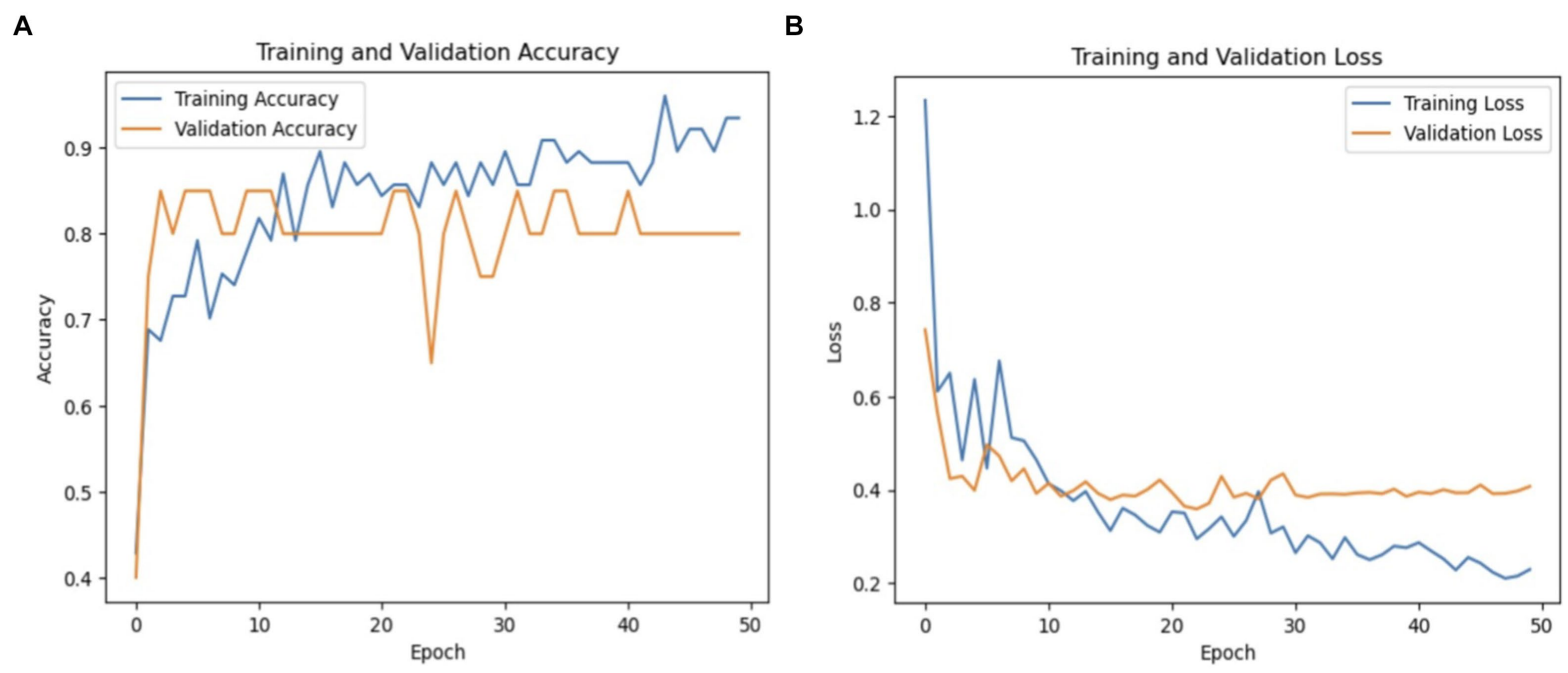
**(A)** Validation and training accuracies of the model, **(B)** model loss of the ResNet50v2 model.

The macro average precision, recall, and F1-score were 81, 77, and 78%, respectively. These metrics underscore the model’s efficacy in distinguishing between PD and healthy individuals, although there remains room for improvement, particularly in increasing the recall for healthy cases. Figure 10 graphically represents the performance of the ResNet50V2 model over 50 epochs.

Figure 10A shows the validation and training accuracies, which improved significantly from 40% initially to 90% for training and 85% for validation by the final epoch, indicating effective learning. Figure 10B illustrates the model’s loss over the training period, with a reduction from a preliminary loss of 1.20 to an ending loss of 0.20 for training and 0.40 for validation, reflecting good enhanced prediction accuracy of the model.

### 4.4 Testing results of the DenseNet169 model

The testing classification results for the DenseNet169 model in detecting PD using spiral drawing images are summarized in Table 9. The DenseNet169 model achieved an overall accuracy of 85%, indicating a high level of performance in distinguishing between PD patients and healthy individuals based on their spiral drawing patterns.

**Table 9:** Testing classification results of the DensNet169 model.

	Precision %	Recall %	F1-score %	Support	Accuracy %
Parkinson	80	100	89	12	**85**
Healthy	100	62	77	8	
Macro average	90	81	83	20	

The model showed 80% precision and 100% recall for Parkinson’s cases. This implies that there were no false negatives in the model’s identification of all actual cases of PD. However, as the precision score shows, the model did generate some erroneous positives. For Parkinson’s cases, the F1-score was 89%, indicating a fair trade-off between recall and precision for this class.

The model’s precision for healthy individuals was 100%, meaning that it was always accurate when it projected a case to be healthy. The recall rate for healthy patients was 62%, indicating that some genuine healthy instances were overlooked by the algorithm, leading to misleading negative results. Compared to the Parkinson’s class, the F1-score for healthy persons was 77%, indicating a reduced but still acceptable balance between precision and recall. The macro averages of 81% for recall, 83% for F1-score, and 90% for accuracy show how well the model performed generally in both classes. The recall macro average shows that there is still need for growth in accurately recognizing every instance across both classes, but the high precision macro average shows how well the model can make positive predictions. Figure 10 shows a graphical representation of the performance plots of the DensNet169 model.

As seen in Figure 10, the training accuracy of the model started at 50% and steadily increased to 89% by the last epoch. Simultaneously, there was an upward trend in the validation accuracy, starting at 60% and reaching 83%. The training loss was reduced significantly from 90 to 20% in terms of model loss. In a similar vein, the validation loss significantly decreased, going from 100 to 55%. Collectively, these indicators show how the model’s performance and capacity for generalization have increased during the training phase.

## 5 Discussion of the results

**Figure 11:**
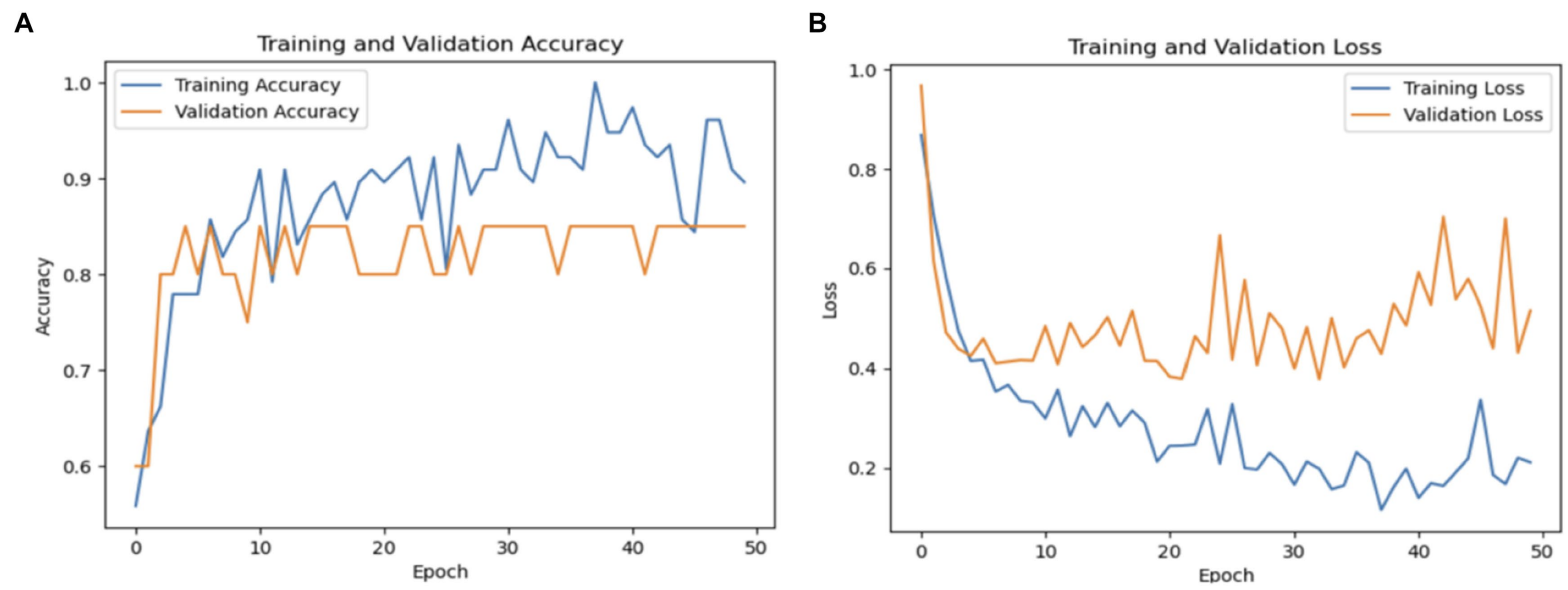
**(A)** DensNet169 model training and validation accuracy and **(B)** model loss.

PD is a neurodegenerative condition that progresses over time and is characterized by both motor and non-motor symptoms. Accurate identification of PD is essential for timely intervention. Conventional diagnostic methods often rely on subjective neurological exams and clinical evaluations, leading to potential inaccuracies. Therefore, there is growing interest in leveraging advanced computational and machine learning methods to enhance diagnostic precision. Figure 11 shows performance of DenseNet169.

In this study, we assessed the performance of several deep learning models VGG19, InceptionV3, ResNet50V2, and DenseNet169 in identifying PD from spiral drawing tests. The results highlight the strengths and limitations of each model. The VGG19 model achieved a total accuracy of 72%, demonstrating the lowest performance in detecting PD cases and a higher rate of false positives and false negatives compared to the other models.

**Figure 12:**
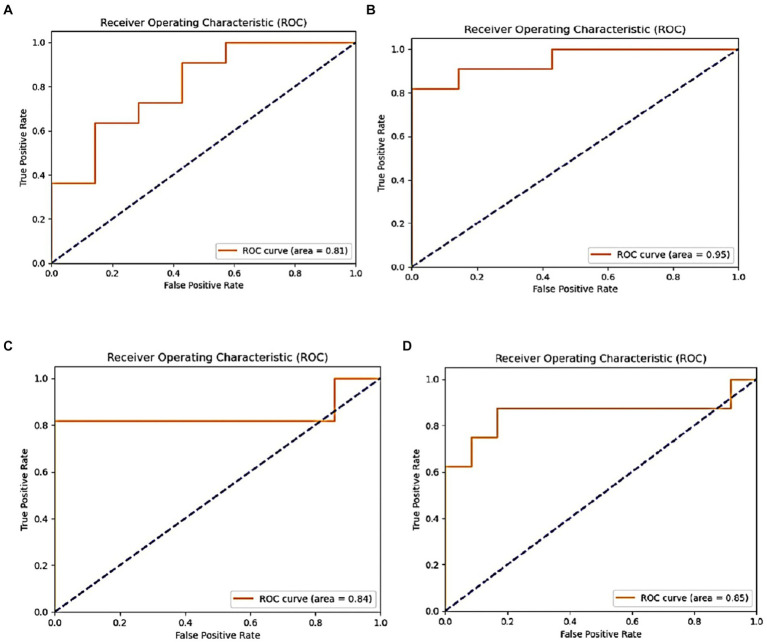
ROC metric of the proposed models: **(A)** VGG19, **(B)** Inception, **(C)** ResNet50v2, and **(D)** DensNet169.

The DenseNet169 model demonstrated an accuracy rate of 85%, whereas the InceptionV3 model achieved a higher accuracy of 89%, both surpassing the performance of the ResNet50V2 model. The InceptionV3 model, in particular, exhibited excellent sensitivity and minimal false positives, making it highly effective in identifying both Parkinson’s disease (PD) and healthy cases. In contrast, ResNet50V2 achieved an accuracy of 80%, with notable precision in identifying PD cases but less efficacy in classifying healthy individuals. Collectively, these findings indicate that transfer learning models based CNN architectures have capability to classify Parkinson’s disease status using intelligent spiral drawings features, especially InceptionV3 and DenseNet169, that showed substantial potential for enhancing PD classification. Future research should focus on optimizing these models further, exploring additional data sources, and validating these findings in real-world clinical settings. Figure 12 displays the ROC of the proposed models, where the InceptionV3 model is found to achieve a high percentage of 91%.

This subsection highlights the variations in accuracy outcomes by providing an analysis of several techniques used on the same dataset of 102 spiral images. The authors reported a 67% accuracy rate using the RF technique in (38). According to Haq et al. (39), lightning CNNs achieved an accuracy of 63.33%, while in Huang et al. (41), a standard CNN approach demonstrated a significant increase with an accuracy of 83%. By comparison, the InceptionV3 model we used in our investigation produced the best accuracy of 89%. This better performance highlights the potential of sophisticated DL architectures above more conventional machine learning and simpler neural network approaches, proving their effectiveness in correctly detecting PD using spiral drawing images. Table 10 displays the comparative analysis between our study results and existing ones based on the same dataset and accuracy metric.

**Table 10:** Comparison of the contribution of the present study with existing research.

Reference ID	Approach	Dataset	Accuracy
Prasuhn et al. (44)	RF	Same dataset 102 spiral images	67%
Rasheed et al. (45)	Lightning CNNs	Same dataset 102 spiral images	63.33%
Pfister et al. (47)	CNNs	Same dataset 102 spiral images	83%
Our study	InceptionV3	Same dataset 102 spiral images	89%

## 6 Conclusion

The timely detection of PD is of utmost significance. The complexity of identifying PD necessitates the development of effective diagnostic instruments. In this work, PDD was determined by examining the Parkinson’s spiral test. In contrast to other investigations in the literature, this study regarded the Parkinson’s spiral test as an issue of recognition. Furthermore, pattern recognition approaches can yield favorable outcomes when used in the analysis of spiral images in PD. This strategy can enhance the effectiveness of diagnosing PD, a condition that is challenging to detect in its early stages. The proposed approach utilized a standardized dataset of 102 spiral samples obtained from individuals diagnosed with PD. The implementation involved the use of VGG19, InceptionV3, ResNet50v2, and DenseNet169 models for the detection of PD utilizing spiral drawings. The aim of this work was to improve the diagnostic process of PD by utilizing transfer learning models. The approach shows promising results in diagnosing PD by analyzing the movement patterns of patients with PD. The classifier, trained on photos of the spiral drawing challenge, achieved an accuracy of 89% and an ROC score of 91% using the InceptionV3 and ResNet50v21 models. The use of DL-based analysis can enhance the efficiency and accessibility of spiral drawing assessment in clinical and research contexts due to its automated and scalable nature. Creating a deep learning system that utilizes spiral drawing images to detect PD can be a valuable method for aiding clinical decision making and advancing drug research. It can improve the diagnostic process, assist in selecting and monitoring patients in clinical trials, and offer objective measures of outcomes, ultimately leading to better patient care and the progress of PD research. The limitation of this research is that it did not investigate the possibility of use spiral drawings to identify other associated movement disorders; instead, it concentrated on utilizing them to create a system for diagnosing PD. The study showed that spiral image analysis is a useful tool for diagnosing PD, but it did not look into whether the technique can distinguish PD from other disorders that can similarly impair motor function, such essential tremor. Another key limitation is that the data utilized was based on previously diagnosed PD participants, thereby making it more challenging to apply this AI approach as PD diagnostic criteria, given that the classification is already known. However, this research demonstrates that more sophisticated transfer learning architectures can improve on previous deep learning approaches for PD classification. As additional study data becomes available, especially spiral drawing data that can be collected in a general population of prodromal PD or those displaying motor symptoms, such architectures can be readily adapted.

Overall, although spiral image analysis for PD classification shows promise in the current research, more investigation is required to examine the approach’s more extensive prospective applications and prove its efficacy for a larger range of movement disorders and patient demographics. Future research addressing these limitations may result in an even more potent and therapeutically valuable tool to aid in the differential classification and early detection of PD and associated disorders. In Future studies will try to solve this issue for improving the system.

## Data Availability

The datasets presented in this study can be found in online repositories. The names of the repository/repositories and accession number(s) can be found below: the dataset is available in this link, https://www.kaggle.com/datasets/kmader/parkinsons-drawings.
